# Controlled sampling of ribosomally active protistan diversity in sediment-surface layers identifies putative players in the marine carbon sink

**DOI:** 10.1038/s41396-019-0581-y

**Published:** 2020-01-09

**Authors:** Raquel Rodríguez-Martínez, Guy Leonard, David S. Milner, Sebastian Sudek, Mike Conway, Karen Moore, Theresa Hudson, Frédéric Mahé, Patrick J. Keeling, Alyson E. Santoro, Alexandra Z. Worden, Thomas A. Richards

**Affiliations:** 1 Living Systems Institute, University of Exeter, Stocker Road, Exeter, UK; 2 Laboratorio de Complejidad Microbiana y Ecología Funcional, Instituto Antofagasta, Universidad de Antofagasta, Antofagasta, Chile; 3 Monterey Bay Aquarium Research Institute, Moss Landing, CA USA; 4 CIRAD, UMR LSTM, Montpellier, France; 5 Department of Ecology, University of Kaiserslautern, Kaiserslautern, Germany; 6 Department of Botany, University of British Columbia, Vancouver, BC Canada; 7 Department of Ecology, Evolution and Marine Biology, University of California, Santa Barbara, CA USA; 8 Ocean EcoSystems Biology Unit, GEOMAR Helmholtz Centre for Ocean Research Kiel, Kiel, Germany; 9 Department of Zoology, University of Oxford, 11a Mansfield Road, Oxford, OX1 3SZ UK

**Keywords:** Water microbiology, Biodiversity, Microbial ecology

## Abstract

Marine sediments are one of the largest carbon reservoir on Earth, yet the microbial communities, especially the eukaryotes, that drive these ecosystems are poorly characterised. Here, we report implementation of a sampling system that enables injection of reagents into sediments at depth, allowing for preservation of RNA in situ. Using the RNA templates recovered, we investigate the ‘ribosomally active’ eukaryotic diversity present in sediments close to the water/sediment interface. We demonstrate that in situ preservation leads to recovery of a significantly altered community profile. Using SSU rRNA amplicon sequencing, we investigated the community structure in these environments, demonstrating a wide diversity and high relative abundance of stramenopiles and alveolates, specifically: Bacillariophyta (diatoms), labyrinthulomycetes and ciliates. The identification of abundant diatom rRNA molecules is consistent with microscopy-based studies, but demonstrates that these algae can also be exported to the sediment as active cells as opposed to dead forms. We also observe many groups that include, or branch close to, osmotrophic–saprotrophic protists (e.g. labyrinthulomycetes and Pseudofungi), microbes likely to be important for detrital decomposition. The sequence data also included a diversity of abundant amplicon-types that branch close to the *Fonticula* slime moulds. Taken together, our data identifies additional roles for eukaryotic microbes in the marine carbon cycle; where putative osmotrophic–saprotrophic protists represent a significant active microbial-constituent of the upper sediment layer.

## Introduction

Marine sediments are microbially driven ecosystems and encompass one of the largest reservoirs of organic carbon on Earth [1–3]. These ecosystems are difficult to access [4, 5] yet marine sediments harbour a large reservoir of eukaryotic microbial diversity [6–10] of unknown function. Osmotrophy [11] and other lifestyles that involve degradation of organic matter are considered important in marine sediments [12]. These processes are responsible for the breakdown of complex compounds that reach the sea floor [3]. Among the eukaryotes, fungi perform key osmotrophic/lysotrophic/saprotrophic (OLS) biogeochemical functions in terrestrial environments, however, the equivalent role remains underexplored in marine sediments [13]. A number of studies have shown the presence of fungal DNA and RNA in marine sediments [9, 14–16] but the diversity of wider OLS protist groups remain untested, even though such groups encode lifestyle strategies often critical for biogeochemical process [12, 13].

An issue for molecular diversity studies is that cell-free DNA and DNA of dead cells can persist over long periods of time, misleading DNA-based analysis. The cold temperatures found at depth (e.g. 4 °C) can also play a role in DNA preservation. For example, the DNA profiles of terrestrial soils can be ‘contaminated’ with sequences from dormant-, dead- or lysed cells [17]. Sequencing of RNA-derived template can circumvent this issue because RNA is an unstable macromolecule and so is thought to represent the active community sampled. The susceptibility of RNA to degradation and the rapid response times of some microbial taxa to changes in environmental conditions make preservation of RNA an important methodological consideration [18]. However, it is currently not possible to minimise time to preservation when sampling at depth, where recovery can add several hours, during which time samples can undergo huge changes in pressure and potentially temperature. These factors must presumably alter both community composition and RNA longevity.

To date, remotely operated vehicle (ROVs push core and scoop approaches) have precluded preservation at depth, despite there being several chemical possibilities for preserving RNA. LifeGuard™ is an RNA preservation buffer which, according to US patent (6,458,546), contains no compounds that induce the precipitation of proteins and organic compounds, which can lead to the drastic loss of nucleic acid molecules or biases in community structure [19]. LifeGuard™ has been successfully employed for environmental RNA sampling, for example from: rainforest surface soils [20] and spring sediments [21, 22]. For marine sediment studies, limitations in the collection devices have prevented RNA preservation at the point of sampling, with samples resuspended in buffer or frozen upon retrieval at the surface [9, 10, 23, 24].

**Fig. 1:**
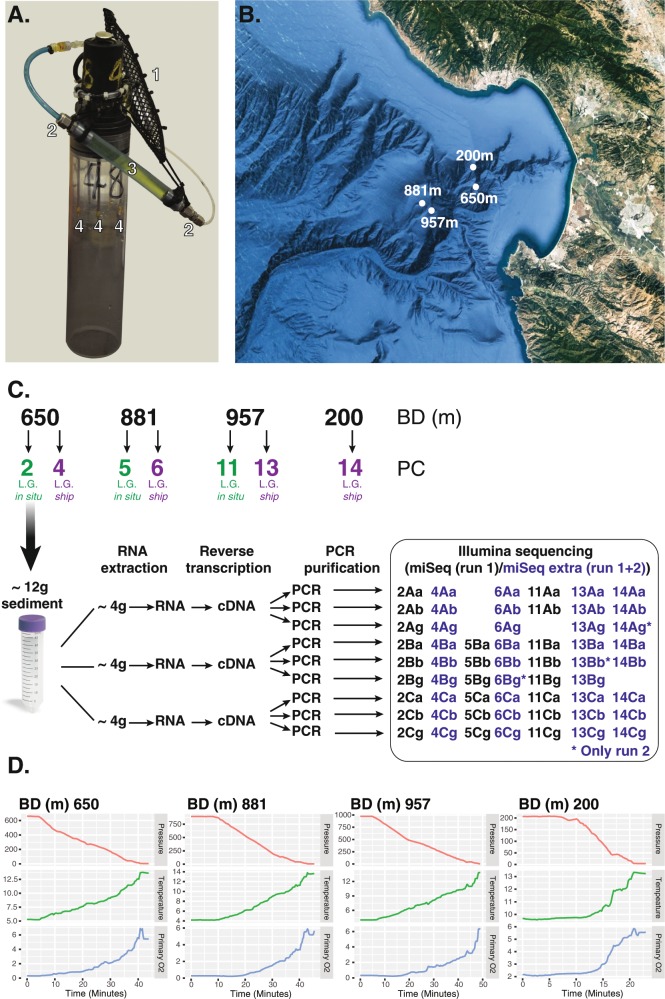
Sampling approach. **a** Photograph of the custom-built multi-needle injector system: 1-buffer reserve bladder, 2-unidirectional valve, 3-tubing allowing for ‘compression pumping’, 4-injector needles with sideways apertures (see Film S1 for operation). **b** Map showing the Monterey Canyon and indicating the bottom depth (BD) of sampling sites (GPS co-ordinates mapped using google earth (http://earth.google.com) provided in the ‘Materials and methods’ section) where sediment push cores (PC) were taken. **c** Sample collection and processing diagram illustrating how the replicate samples were processed for RNA extraction. These samples were coded in the following manner, (i) biological replicates were distinguished: A, B, and C. (ii) Independent reverse transcription PCR replicates equivalent to technical replicates were distinguished: a, b and g. Numbers 2-4-5-6-11-13-14 indicate the core the samples came from. Asterisk indicates samples with reads only from our extended sequencing effort, i.e. run 2. **d** Abiotic profile change during ROV recovery recorded for four separate core sample sets: pressure (decibars), temperature (C°) and primary O_2_ (ml/l).

To tackle this problem, we have developed a tool for the injection of preservation buffer into sedimentary samples at the point of sampling (Supplementary Film S1 and Fig. 1a). Using this approach, we collected in situ injected and surface-preserved samples from three sets of marine sediments. To compare these sampling approaches, we sequenced the V4 region of the eukaryotic SSU rRNA gene from cDNA reverse transcribed from total RNA. We demonstrate a significant difference in community sampling trends between the two approaches. Using these data, we investigate the putative phototrophic and OLS eukaryotic groups present in the sediment samples. These results suggest a hitherto underexplored OLS protistan community in marine sediments and implicate a diverse range of ribosomally active eukaryotic microbes in multiple aspects of the marine carbon cycle in the surface sediments.

## Materials and methods

### Study site and sampling approach

Pilot experiments were conducted using a standard push core (23 cm core tube, 7 cm inner diameter) modified as a reagent injector (Fig. 1a) with 5 cm needles (with side-on apertures). The ‘initial cruise’ was conducted on the R/V Pt. Lobos using ROV *Ventana* (8th September 2011, Dive number V3640, location 36° 73′ N, −121° 93′ W, depth 97 m). This ‘pilot cruise’ site was 12 km from the 200 m site and 22 km to the 881/957 m sites (see below). All these sites had similar sedimentary characteristics. The injector was deployed by an ROV preloaded with PBS-containing fluorescein (Sigma-Aldrich, 0.3 mM, final concentration) and 0.75 μm YG fluorescent-polystyrene beads (Polysciences Inc.). The buffer was pumped into the sediment cores using the ROV manipulator claw (taking 1–2 min) to test buffer penetrance. Follow-up microscopy demonstrated the injected buffer saturated the top 1–2 cm layer of the sediment core. All further sampling was limited to the top 1 cm of the core.

Prior to, and after each use, the injector was set up/decommissioned in a laminar flow hood, with the system repeatedly washed through with 70% ethanol, with all liquid expelled before being loaded with sampling buffer. The system was then fully discharged, the flow-through sample buffer discarded and then reloaded with new sampling buffer. The reserve bladder and injector pump were filled using sterile syringes with a mix of 5 mg of fluorescein dissolved in 100 ml of LifeGuard™ (MoBio). The system was then primed prior to deployment so that ~5 ml of buffer was released from the needles recovered and stored at −80 °C for SSU rRNA gene analysis as a control for contamination.

Samples for diversity analyses were collected in September 2011 using the ROV *Doc Ricketts* from the North Pacific Ocean (Monterey Bay) at 200 m (36° 47′ N, 122° 3′ W), 650 m (36° 45′ N, 122° 3′ W), 881 m (36° 43′ N, 122° 11′ W) and 957 m (36° 42′ N, 122° 10′ W) depth (Fig. 1b). The samples were not methane super-saturated. Push core and injector set-up was conducted using sterile gloves and apparatus. Two approaches were used: (i) *LG-in situ*; injection of LifeGuard™ in situ using the injector system (Fig. 1a and Film S1) followed by suspension in additional LifeGuard™ at the surface after sample recovery (following the manufacturer’s instructions), and (ii) *LG-ship*; samples without in situ injection were recovered and resuspended on board the ship in LifeGuard™ (following the manufacturer’s instructions). The paired cores with the different treatments were each deployed within 1 m of each other.

During preparation and ROV recovery, samples were taken to control for potential contamination introduced during set-up and/or ROV operation. Specifically, 5 ml of buffer was recovered from the wash through as the system was primed (described above) and another 5 ml of un-injected buffer sample was recovered from the buffer reserve bladder after deployment. These control samples were immediately frozen at –80 °C. Across the three needle-injected samples 10 (650 m sample), 30 (881 m) and 20 ml (957 m) were left in the bladder/system after deployment indicating that between 65 and 85 ml were injected into 1–2 cm top layer of the sediments. On ship, ~12 g of surface sediment taken from around the needle insertion points from each sampling core was transferred immediately to a 50 ml centrifuge tube, using a sterilised spatula in a laminar flow hood. Additional LifeGuard™ solution was added (3 ml of additional buffer per 1 g of sediment), shaken vigorously and stored at –80 °C and then shipped on dry ice to the UK. We note this process may fail to sample some eukaryotes lysed by the process of sampling and for which the RNA molecules of lysed cells were released into the water column space of the core or the resuspension buffer during preservation.

### RNA extraction, cDNA synthesis and SSU V4 PCR

Samples were defrosted on ice and centrifuged at 2500 × *g* for 5 min at 4 °C to remove supernatant. Each push core sample (Fig. 1c) was divided into three sub-samples of ~4 g sediment (A, B and C) and processed individually using the MoBio PowerSoil total RNA kit with the following variations: (i) 50 μl of SR7 solution was used to resuspend the RNA, (ii) the SR4 solution incubation was conducted at −20 °C, (iii) to improve lysis of ‘robust’ microorganisms a freeze-thaw cycle followed by physical disruption using silica carbide beads was added to the C1 buffer step of the protocol. The DNase treatment was performed following the manufacturer’s protocol. RNA quality and quantity was checked using a 2100 Bioanalyzer (Agilent technologies). Each RNA sample was then tested for DNA contamination by PCR amplification using 18S (V9 region: 1380F 5′-CCCTGCCHTTTGTACACAC-3′ and 1510R 5′-CCTTCYGCAGGTTCACCTAC-3′) and 16S (V6 region: 967FPP 5′-CNACGCGAAGAACCTTANC-3′ and 1046RPP 5′-CGACAACCATGCANCACCT-3′) primers. PCR reactions were performed in 25 μl reactions, using Phusion polymerase with 1× GC buffer, 0.35 μM each primer, 200 μM of each dNTP and 3% DMSO. The 1380F/1510R, PCR reactions consisted of an initial denaturation step (98 °C for 30 s), followed by 30 cycles of 98 °C for 30 s, 57 °C for 60 s and 72 °C for 90 s, before a 10-min extension at 72 °C. The 967F-PP/1046R-PP PCR reactions consisted of an initial denaturation step at 98 °C for 30 s, followed by 30 cycles of 98 °C for 30 s, 60 °C for 45 s and 72 °C for 60 s, before a 10-min extension at 72 °C (no discernible PCR band compared with positive controls were identified on a 1% agarose gel). Each RNA sample was reverse transcribed into cDNA using SuperScript III reverse transcriptase (Invitrogen, UK), adding 0.3–1 µg of RNA template along with random primers from the kit (manufacturer’s instructions). The resulting cDNA was quantified with a Qubit fluorimeter using an ssDNA kit (Invitrogen) and diluted to a final concentration of 10 ng/µl.

Unspent reagent samples were collected before and after deployment as control samples, as described above. RNA and DNA extractions were conducted from these samples using the PowerWater RNA and DNA Isolation Kits (MoBio) and analysed using a Bioanalyzer, which demonstrated the absence of clear RNA or DNA signal. These control samples were then subject to PCR amplification using 18S-V9 and 16S-V6 primers and PCR protocols as outlined above. These were both negative (i.e. no discernible 18S or 16S band present compared with positive controls). The RNA samples were then reverse transcribed and PCR amplified using 18S rDNA primers (as above) and again the PCR reactions were negative.

For each cDNA sample three PCR amplifications were conducted using the eukaryotic V4 SSU primers: TAReuk454FWD1 (5′-CCAGCASCYGCGGTAATTCC-3′) and TAReukREV3 (5′-ACTTTCGTTCTTGATYRA-3′) [25]. PCR reactions were performed in 25 µl reactions, and contained 10 ng of cDNA template, 1× Phusion GC buffer, 0.35 µM of each primer, 200 µM of each dNTP, 3% DMSO and 0.5 units of Phusion^®^ High-Fidelity DNA Polymerase (BioLabs). PCR reactions consisted of a denaturation step at 98 °C for 30 s, followed by 10 cycles of 10 s at 98 °C, 30 s at 53 °C and 30 s at 72 °C, and then 15 cycles of 10 s at 98 °C, 30 s at 48 °C and 30 s at 72 °C, and an elongation step at 72 °C for 10 min. Amplicons were checked on 1% agarose gels for amplification and purified using a GeneJET PCR Purification Kit (ThermoFisher), eluted in 25 µl of elution buffer and quantified with a Qubit dsDNA HS kit (Invitrogen).

### Library preparation and sequencing

Illumina sequencing libraries were generated for each amplicon library using NEXTflex DNA sequencing kits with NEXTflex DNA barcode with eight-base indices (Bioo Scientific). Each library was subject to a 300 bp paired-end sequencing run on an Illumina MiSeq 2500 platform (run 1) at the University of Exeter Sequencing Service. All samples were pooled on the same ‘lane’, so that any trend identified between sample sets was not the product of between-lane sequencing efficiency/dynamics. All the cores with LifeGuard™ added at the surface (*LG-ship)* (4, 6, 13 and 14) were sequenced for a second time using one lane of Illumina MiSeq (run 2) (Fig. 1c). Sequences are available at the NCBI SRA, accession numbers: SAMN11051173-204 (Bioproject PRJNA521526).

### Quality filtering, selection of operational taxonomic units (OTUs) and taxonomic assignment

Raw data from the Illumina libraries were assigned to individual samples by their barcodes. Merged (overlapped) paired-end reads were created using PEAR [26] and any un-merged reads were excluded. Cutadapt was then used to remove primer sequences and for quality control [27] using default parameters, de-replication was performed using VSEARCH [28]. Processed amplicons were clustered into OTUs using SWARM v2 [29]. Taxonomic assignment was accomplished by taking the most numerous sequence from each OTU as a search seed for STAMPA (https://github.com/frederic-mahe/stampa) against PR2 V4 SSU rRNA database (version gb 203) [30]. Chimeric sequences were identified and removed using UCHIME [31] implemented in VSEARCH. Code information is available at https://github.com/frederic-mahe/swarm/wiki/Fred’s-metabarcoding-pipeline. Only OTUs assigned as eukaryotic were kept for further analysis; singletons and OTUs present in a single sample were excluded.

### Diversity analysis and community composition

Alpha- (within sample) and beta- (among samples) diversity analyses were conducted on both sample types from run 1 (*LG-in situ* and *LG-ship*). Any OTUs assigned as Metazoa (19% of the reads) were excluded. The Shannon, Chao1 and PD calculation methodology include a default rarefaction approach and so were calculated using the QIIME v1 scripts (alpha_rarefaction.py) resulting in 18,564 reads per sample. An analysis of variance (ANOVA) was conducted to test for group differences using R [32].

Species (OTUs) abundance distribution and species (OTUs) accumulation curves, based on the number of reads, were compared between treatment groups using R (ggplot2 library [33]). For this approach we had to rarefy the dataset using a different approach (QIIME v1 scripts-single_rarefaction.py). For individual sample comparison the data were rarefied to 19,454 reads, for samples pooled by core the data were rarefied to 473,271 reads.

For the beta-diversity, Bray Curtis distance matrices from a relative abundance and a presence/absence (Sorenson’s index) OTU table were performed using R (Vegan library). Unweighted and weighted UniFrac [34] distance matrices were calculated using QIIME v1 scripts. The phylogenetic tree was calculated using FastTree2 using the ‘make_phylogeny.py’ QIIME v1 script using the sequences aligned with MAFFT v7 [35]. The diversity profiles were represented as a non-metric multi-dimensional scaling (NMDS) ordination using the MASS package [36] in R [32]. Clusters based on Bray Curtis and UniFrac distances were generated using hierarchical clustering (UPGMA) using the hCLUST function in R.

As mentioned above, the *LG-ship* samples were subjected to additional sequencing (run 2) to further explore the diversity profiles. Runs ‘1’ and ‘2’, were pooled by sediment core and normalised (473,589 reads—as the defined minimal threshold across all replicates with run 1 and 2 combined). Stacked bar charts were generated with the reshape2 and ggvis libraries in R to identify taxonomic composition of these samples.

### Group specific phylogenetic analysis

SSU phylogenetic trees were calculated for the OTUs identified from runs ‘1’ and ‘2’ combined. We limited our phylogenetic analysis to OTUs that were composed of ≥20 reads and so these results are limited to OTU groups with increased relative representation. The resulting trees encompass a large amount of sequence diversity, yet are derived from a short alignment of the ~400 bp V4 region of SSU rRNA gene, resulting in a highly skewed data matrix with relatively few informative alignment positions but very many sequences. This in part explains why many of the nodes recovered in the phylogenies have low bootstrap values. To construct the different phylogenies, published reference trees of known OLS eukaryotic groups (Stramenopiles [37], Labyrinthulomycetes [38] and Opisthokonts [39]) were combined with the ≥20 sequence OTUs. Sequences were aligned using MAFFT v7 and manually checked with SeaView (Version 4.5.2). Maximum Likelihood (ML) phylogenetic trees were inferred using RAxML HPC2 [40] in the CIPRES gateway under the GTRGAMMA model with 100 rapid bootstrap replicates [41].

## Results

### Variance in the community structure identified across replicates and treatments

**Fig. 2:**
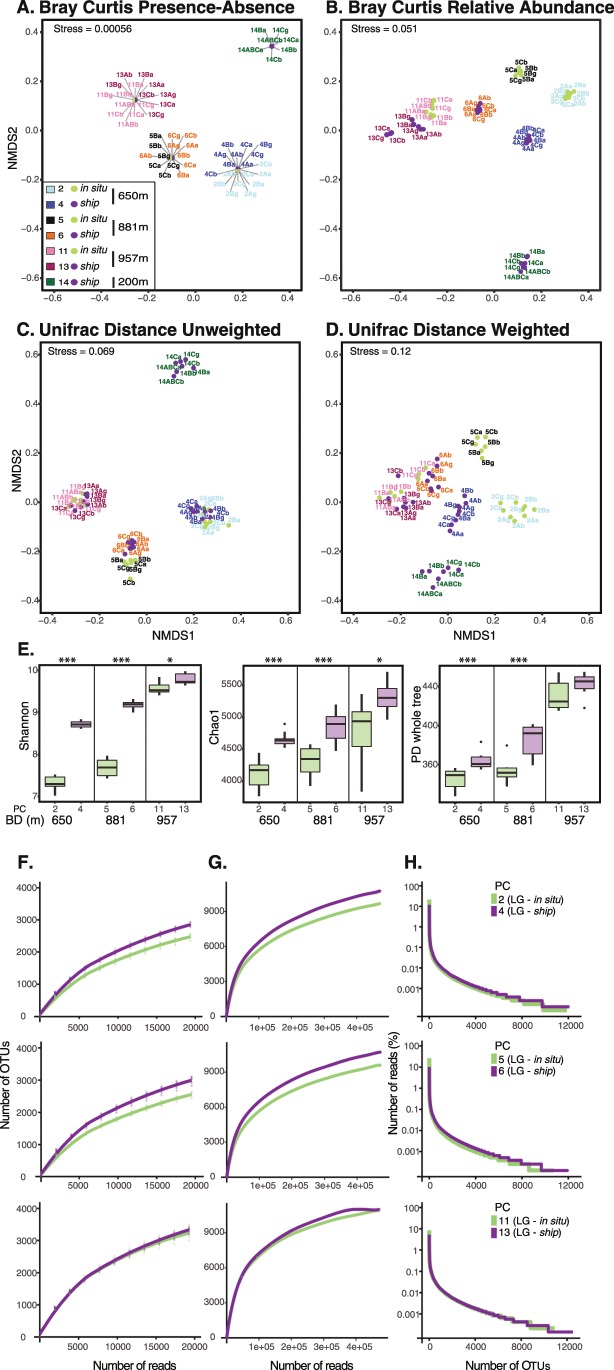
Descriptive characteristics of the diversity profiles recovered from amplicon sequencing with and without in situ RNA preservation. **a**–**d** Non-metric multi-dimensional scaling (NMDS) ordination plots comparing push cores/treatments and sample replicates. NMDS plot derived from Bray Curtis distance based on; **a** OTU Presence–absence data (lines used to label overlapping dots), and **b** OTU relative abundance data. NMDS plot derived from Unifrac distance analysis of OTU composition based on; **c** Unweighted (qualitative) comparisons, **d** Weighted (quantitative) comparisons. OTUs are defined by swarm analysis (see ‘Materials and methods’). Unifrac encompasses a distance measure using sequence based phylogenetic information to compare samples. It is possible that due to the similar provenance of the sampling cores, the OTU constellation recovered from each core/sample partially lacked phylogenetic resolution, and so these comparisons did not generate a significant difference when using Unifrac distance measures, therefore reducing the resolution and blurring the distinction between different core/treatment samples. **e** Comparisons of median Alpha diversity indices showing significant differences between injected (*LG-in situ*; green boxes) and non-injected samples (*LG*-*ship*; purple boxes) in eight of the nine comparisons. (*p < 0.05, ***p < 0.001). The boxes show the first and third quartiles. The upper and lower whiskers extending from the box show the most extreme value that is within 1.5× of the interquartile range. **f** Species (OTUs) accumulation loess curves from samples studied separately (threshold 19,454 reads). Smooth dots are showing all individual samples. **g** Species (OTUs) accumulation loess curves from samples pooled by core (threshold 473,271 reads). Smooth dots are showing all individual samples. **h** Species (OTUs) abundance distribution curves from samples pooled by core (threshold 473,271 reads) based on the number of reads. Key differences here are shown between the most abundant OTUs sampled via the two different methods. BD bottom depth, PC push core.

Sediments from a 7-cm-diameter section taken from each push core, for a total of seven cores from multiple sites (see ‘Materials and methods’), were divided into three equivalent portions. These were considered separate biological replicates. The SSU PCR was conducted in triplicate for each cDNA sample (technical replicates). In the best cases, we generated nine replicates per core. In a subset of cases some steps failed, limiting sampling to six or more replicates (Fig. 1c). After bioinformatic processing and rarefaction analysis, every technical replicate contained a minimum of 19,454 sequence reads, while each biological replicate, encompassing multiple technical replicates, ranged from 71,268 reads (run 1) to 1,050,451 (run 1 and 2 combined) (Table S1). The beta-diversity analyses demonstrated high clustering similarity between replicates (Fig. 2a–d). UPGMA dendrograms calculated using the Bray Curtis distance (presence/absence and relative abundance, respectively) resulted in a similarity range of 57.1–61.9% and 73–83.2% for the technical replicates and 54.1–57.8% and 64.9–75.8% for the biological replicates (Fig. S1a, b), demonstrating similar community profiles from samples of similar provenance.

### Effect of RNA preservation approaches on diversity/abundance profiles

For three of the sampling depths (650, 881 and 957 m) two cores were recovered, one *LG-in situ* and one *LG-ship*. For the fourth set of samples at 200 m, the injection system failed and only *LG-ship* samples were recovered. For the 200 m, 650 m and 881 m depth samples, it took 17, 40 and 37 min to recover the samples, respectively. Due to the depth, tide and rough sea state, the 957 m depth sample took 127 min to recover. Abiotic parameter tracking demonstrated considerable changes in pressure during ROV recovery, while temperature changes of around 6–10 °C occurred largely in the last ¼ (5–20 min) of the ROV recovery journey (Fig. 1d).

An aim of this study was to identify if *LG-in situ* treatment resulted in a different abundance/diversity profile compared with the paired *LG-ship* samples. After normalising the sampling effort, alpha diversity analyses (Shannon, Chao1 and phylogenetic diversity) (Fig. 2e), species accumulation curves (technical replicates (Fig. 2f) and pooled by core (Fig. 2g)) and species abundance distribution curves (Fig. 2h) were compared. In all three sample-pairs the *LG-in situ* samples demonstrated two altered factors: (i) a lower total number of OTUs with equivalent sequencing efforts and (ii) the abundant OTUs contained higher relative representation (Fig. 2h). This result is mirrored in the accumulation curves, where the *LG-in situ* samples show improved saturation (Fig. 2f, g). Furthermore, the median of the diversity indexes with *LG-ship* (Cores 4, 6 and 13) is higher than the median with *LG-in situ*, indicating a significantly increased diversity profile identified in non-injected samples in eight of nine ANOVA tests (*p* < 0.05, Fig. 2e). Taken together these results show an effect of the *LG-in situ* protocol and demonstrate that injection significantly alters the community profile identified. It is possible that this altered sampling skew could be the result of different levels of RNA preservation efficacy in situ across different taxa. However, this is only testable with large-scale culture-based experiments and is currently not possible as none of the protist taxa sampled in these environments are in culture.

### Comparison of community profile composition across samples

NMDS plots (Fig. 2a, b) of the Bray Curtis distance show sequence-amplicon profiles from the same depth (*LG-in situ* and *LG-ship*) group together, forming clusters of 50.3–51.6% of similarity from the presence/absence analysis (Figs. 2a and S1a) and of 52–56.5% of similarity from the relative abundance analysis (Figs. 2b and S1b). Samples from the same depth are grouping by core/treatment with a higher similarity in the Bray Curtis relative abundance profile (Figs. 2b and S1b, i.e. 64.9–75.8%) in comparison with the presence–absence profile (Figs. 2a and S1a, i.e. 54.1–57.8%). This is because the presence–absence NMDS analysis identified a very similar community across the two different sampling treatments, while the relative abundance NMDS profile shows distinctly different patterns with clearer separation for the different sampling treatments. This result is consistent with the hypothesis that an altered relative abundance pattern is the primary effect of *LG-in situ* treatment. Unweighted (qualitative) and weighted (quantitative) Unifrac distances were also analysed (Fig. 2c, d), giving a similar pattern as identified in the Bray Curtis NMDS analysis (Fig. 2a, b), but showing reduced clustering of some sample replicates, since depth specific groupings were not resolved in the weighted Unifrac distance analysis (Fig. 2d). In terms of specific changes of taxonomic groups between *LG-in situ* and *LG*-*ship*, only the Bacillariophyta (diatoms), a highly abundant group, showed a consistent and strong pattern of change between *LG-in situ* and *LG-ship* sampling strategies (Table S2).

### Eukaryotic microbial community composition of marine sediments sampled

**Fig. 3:**
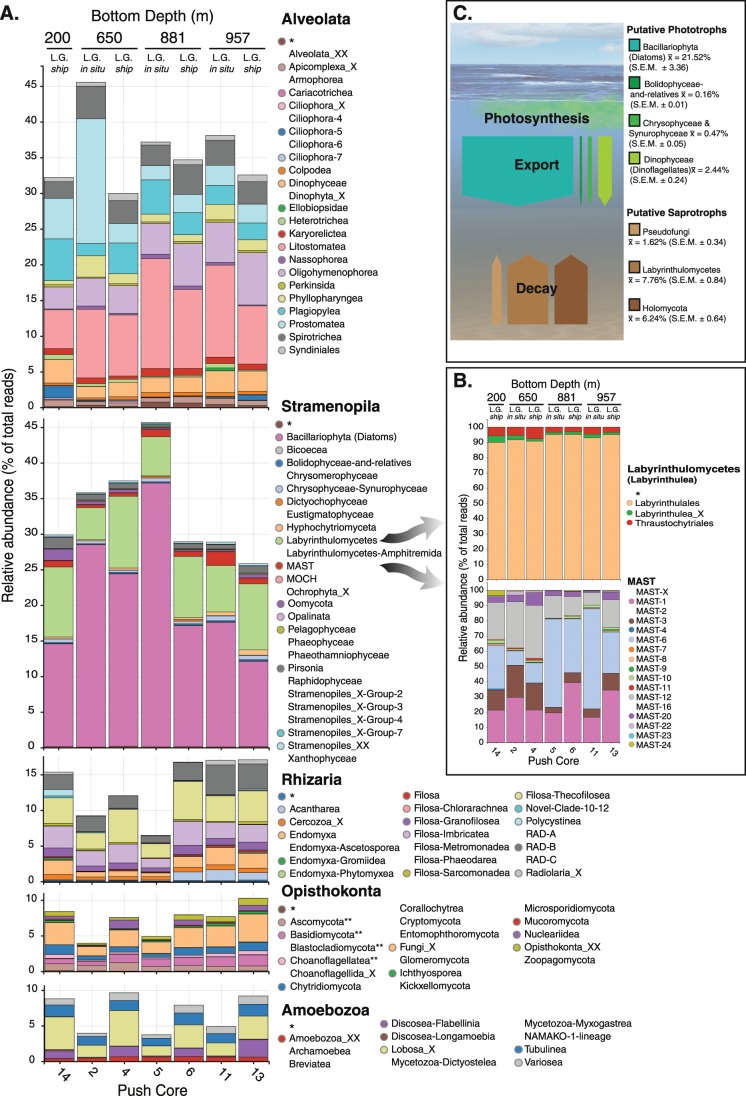
Stacked bar chart showing relative abundance composition from a combination of sequencing run 1 and 2. **a** Representation of the most abundant microbial eukaryotic groups classified by ‘Class’ division using PR2 V4 SSU rRNA database assignation (including all groups with relative abundance >5%) and **b** representation of the MAST and labyrinthulomycetes groups detected. Reads from Runs ‘1’ and ‘2’ were pooled by sediment core and normalised. All groups that appear in the colour coded key were detected in the study but some of them had a low relative quantity (<0.06%) so are not visible in the bar chart and so their colour code circle is removed. Asterisk symbol indicates OTUs affiliating with the wider taxonomic group but of uncertain Class designation. Taxon groups appended with _X (etc.) represent uncharacterised phylogenetic groups that branch with the assigned taxon in the PR2 database classification. **c** Schematic summary figure showing % active rRNA SSU amplicon contribution from putative phototrophic and osmotrophic–lysotrophic–saprotrophic eukaryotes. Holomycota is describes as one unit given that the initial classification of many of these amplicons was initially as fungi but later phylogenetic analysis indicates that they likely branch as fonticulida.

To further investigate the diversity, we performed additional sequencing of one core from each depth of the *LG-ship* samples. We conducted this extended sampling on the *LG-ship* samples because these samples showed the wider diversity profiles. This sequencing effort resulted in a total of 12,555,051 reads (OTU table available via Figshare, 10.6084/m9.figshare.8000492). An overview of the taxonomic composition of the most abundant groups detected in these sediments is shown in Fig. 3a (relative abundance >5%, ‘Class’ divisions). Similar to many marine environments, the alveolate and stramenopile supergroups dominate the diversity profiles [42–44], with both groups showing ~40% relative abundance. However, within the alveolates, the groups that dominate the sediment sample are very different from those observed in water column samples. Rather than a dominance of dinoflagellates and syndinians, including MALVs, the most highly represented alveolates are ciliates, especially the classes Prostomatea and Litostomatea, as well as Spirotrichea and Plagiopylea, consistent with previous analyses [23].

Within the stramenopiles, the ochrophyta Bacillariophyta (diatoms) are the dominant RNA-derived signature. Indeed, of the phytoplankton groups, only the diatoms show high relative OTU abundance (21.52% S.E.M. ± 3.36) confirming that ribosomally active and likely intact diatom cells are exported to the sediments [9, 14, 45] (Fig. 3a–c and Table S2). Additional heterotrophic stramenopiles were also evident in the sediments; Labyrinthulomycetes (Fig. 3a), with a predominance of the labyrinthulales and thraustochytriales (Fig. 3a, b). OTUs assigned to the polyphyletic group known as MArine STramenopiles (MAST) [44] were the third most abundant stramenopiles (i.e. MAST groups -1, -3, -6, and -12 (Fig. 3b)), also consistent with previous studies [37].

The Rhizaria also demonstrated a high relative abundance of transcribed SSU rRNA sequences, with relative abundance of 10–20% across the samples (Fig. 3a). Within the Rhizaria the most abundant classes are the cercozoans: Filosa-Thecofilosea, Filosa-Imbriatea and the uncultured radiolaria group RAD-B [46]. Finally, Opisthokonta and Amoebozoa were also recovered with a relative abundance of ~5% (Fig. 3a). Within the sequence amplicons classified as opisthokonts, a significant portion of the OTUs were tentatively assigned as ‘undefined Fungi’, although resolution of SSU rRNA OTUs within this area of the eukaryotic SSU phylogeny can be misleading (discussed below).

### Phylogenetic diversity of osmotrophic–lysotrophic–saprotrophic eukaryotes

Next, we aimed to investigate the diversity of OTUs putatively branching with OLS taxa. These classifications were based on STAMPA analysis against a PR2 rRNA database [30], followed up with phylogenetic analysis. The eukaryotic groups of interest include: fungi, non-metazoan holozoa such as *Corallochytrium* [47] and the osmotrophic stramenopiles, e.g. pseudofungi [48], thraustochytriales and labyrinthulales [49, 50]. In addition, we also investigated the MAST groups, including MAST-6, which have been shown to be present in marine sediments [37, 51] but for which the trophic function cannot currently be inferred based on phylogenetic position.

### Ribosomally active stramenopile lineages

**Fig. 4:**
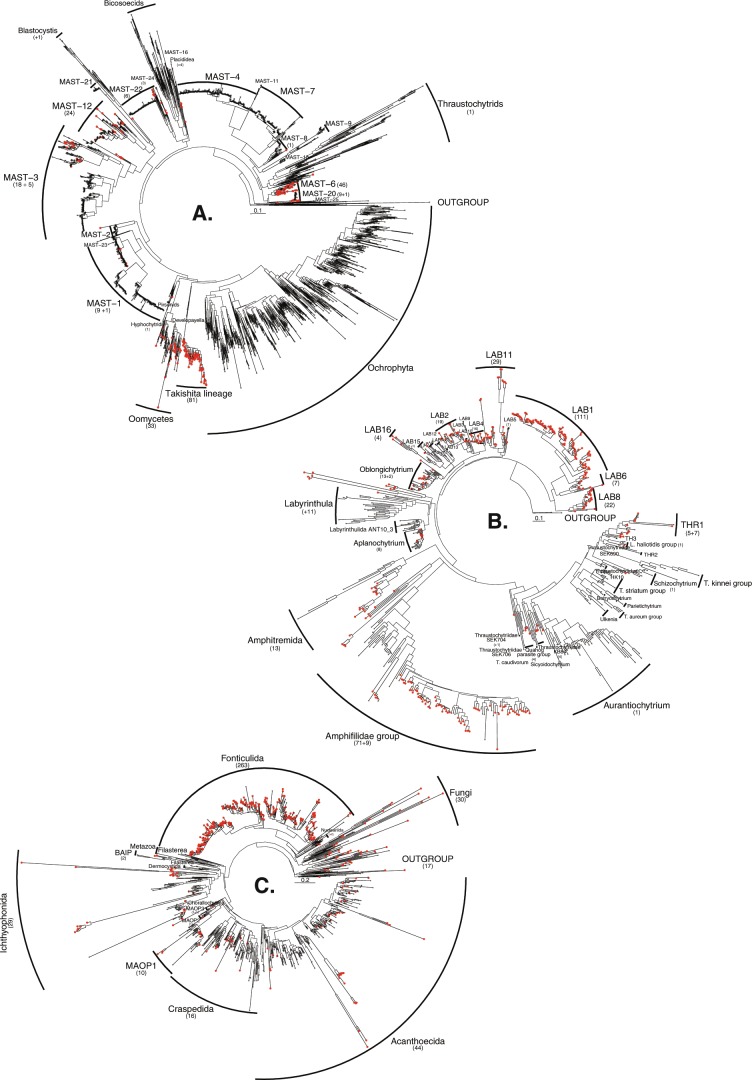
Maximum likelihood phylogenetic trees from a combination of sequencing run 1 and 2. **a** Stramenopile tree. This phylogeny was constructed from the reference tree [37] and is calculated from the V4 region of the SSU rRNA gene and includes 244 OTUs assigned as MAST, Oomycota and Hyphochytriomycota (shown in red). **b** Labyrinthulomycete (Labyrinthulea) tree. This phylogeny was constructed from the reference tree [38] and is calculated from the V4 region of the SSU rRNA gene and includes 365 OTUs (shown in red) assigned as Labyrinthulomycetes. **c** Opisthokonta tree, excluding metazoan sequences. This phylogeny was constructed from the reference tree [39] and is calculated from the V4 region of the SSU rRNA gene and includes 414 OTUs (shown in red) assigned as Opisthokonta (excluding Metazoa, which constituted 75% of the opisthokont reads). Numbers between brackets are the number of newly sampled OTUs included in the group; an additional number is shown when some OTUs branch close to this group but do not branch within the established clade. Only OTUs with >20 sequencers were included here.

OTUs with ≥20 reads classified as MAST or as pseudofungal were incorporated into a stramenopile phylogeny [37] (Fig. 4a). This analysis significantly expanded the known diversity of many of these groups. Specifically, MAST-6 (Figs. 4a and S2) was previously only represented by 7 GenBank SSU sequences and 17 ‘OTU_97_ pyrotags’ [37], yet the OTUs detected here encompassed 46 OTUs with three (OTU-478, -450 and -856) showing high relative abundance (>1000 reads). We also detected significant additions, in terms of V4 tags, for a number of MAST clades, including: MAST-20 (9-OTUs), MAST-22 (6), MAST-3 (18), MAST-12 (24) and MAST-1 (9).

For the pseudofungi groups, we identified an additional 33 OTUs within the oomycete clade (Figs. 4a and S2); 31 of these OTUs grouped with known oomycete sequences [30] with ≥90% bootstrap support. This phylogenetic analysis also identified 81 OTUs that clustered with a clade previously composed of two variant SSU rRNA sequences and described as a novel stramenopile lineage branching near the pseudofungi [23] (Figs. 4a and S2—‘Takishita lineage’). Two of the OTUs identified here appear to have a high relative abundance with >1000 reads. These data suggest this group encompasses significant phylogenetic diversity branching close to *Developayella* [23, 52].

Labyrinthulomycete (Labyrinthulea) OTUs with ≥20 reads, were integrated into a labyrinthulomycete phylogeny [38, 53] (Figs. 4b and S3), demonstrating that the majority of the OTUs identified grouped within the LAB-1 clade, initially named uLa1 [53], and previously described as consisting of five SSU rRNA phylotypes from two sediment samples [53]. The 111 OTUs identified group with LAB-1 with a bootstrap support value of 99%, with 25 OTUs demonstrating high relative abundance (>1000 reads, e.g. OTU6, with 63,933 reads, Fig. S3). Similarly, our results identified significant phylogenetic diversity that is putatively assigned to LAB-6, LAB-8, LAB-2, LAB-4, LAB-11 and Oblongichytrium. Both LAB-8 and LAB-4 groups included OTUs with a high relative abundance (>1000 reads, e.g. LAB-8; OTU-204, -64, -374 and LAB-4; OTU-254). Amplicon sequencing also demonstrates that the labyrinthulomycete Amphifilidae group is very diverse in the sampled environments, with 71 newly identified OTUs branching within the clade with 84% bootstrap support. Two Amphifilidae OTUs (OTU-318 and OTU-319) encompassed >1000 reads (Fig. S3).

### Ribosomally active opisthokont lineages

OTUs with ≥20 reads assigned as opisthokonts (excluding Metazoa, which constituted 75% of the Opisthokonta classified reads) were incorporated into a published tree [39] (Figs. 4c and S4). Initial OTU taxonomic assignment analysis suggested a large number of the OTUs were classified as fungi, or subgroups within the fungi, e.g. Chytridiomycotina or Mucoromycotina (Fig. 3a). However, in the phylogenetic analysis, 263 of these OTUs initially classified as fungi grouped with the fonticulida. Marine fonticulids have previously been described as a highly diverse sister group to *Fonticula alba* [54] and have been consistently recovered from oxic sediments [39]. This grouping was recovered with 56% bootstrap support, and 80% bootstrap support for a clade encompassing 99 of the OTUs detected here together with sequences from other studies [39]. In general, OTUs for this group were abundant across the cores sampled (Fig. S4) suggesting a significant diversity of ribosomally active fonticulids in these marine sediments. Furthermore, 29 OTUs branch within the Ichthyophonida (a separate opisthokont lineage previously described as free-living saprotrophs or parasites of fish [55]), suggesting these sediments harbour a diversity of these enigmatic protists (Figs. 4c and S4).

The phylogenetic analysis demonstrated only 30 OTUs were placed within the Fungi clade and none of these OTUs showed a high relative abundance (Figs. 4c and S4). Interestingly, this diversity included multiple OTUs putatively annotated as ‘chytrid’ fungi (those forming flagellated zoospores) consistent with other reports of chytrids present in marine sediments [15, 56]. However, we note that the phylogenetic placement of these OTUs was poorly supported and many of these OTUs formed long branches.

## Discussion

### Implications of in situ RNA preservation on the community profile detected

Here we show that a protocol modified to allow for in situ RNA preservation leads to a significant and consistent difference in the rRNA community profiles recovered. Specifically, *LG-in situ* samples contained an increased representation (read-count) of the abundant OTUs. Linked to this skew in sampling, we also identified a reduction in the breadth of the diversity profile recovered in *LG-in situ* samples. This effect is significant (Fig. 2e) and is reflected in the shape of the rank abundance and accumulation curves (see Fig. 2f–h). Furthermore, the NMDS analysis based on the Bray Curtis distance matrices showed the *LG-in situ* samples demonstrated an increased variation in the relative abundance profile compared with the presence-abundance NMDS, which in turn was different to that seen in the *LG-ship* samples. These results indicate that the primary effect of fixing RNA-activity is reflected in the community relative abundance profile.

We discuss two possible explanations for this observed shift in community sampling. First, during sample recovery, it is likely that unfixed-RNA is degraded stochastically, and therefore the most abundant OTUs with the largest representation of RNA molecules would see the largest number of losses during partial RNA degradation. Reduction in the RNA representation of the abundant OTUs would therefore free-up sequencing capacity, resulting in an increased diversity profile. This explanation assumes that loss is random and occurs at the same rate across groups.

Second, the *LG-in situ* protocol fixes, or partially fixes, the community at the point of sampling. In contrast, the *LG-ship* treatment allows for community change. As such the variation in the community profile identified could be a product of community change during sample recovery. This possibility is consistent with observations of bacterial community diversity profiles from marine waters [57, 58]. Indeed, comparisons of fixed water column samples with unfixed samples identified distinct changes in meta-transcriptome profiles, suggesting that changes in environment during sampling has an effect on gene expression [59]. Figure 1d shows abiotic parameters during sample recovery demonstrating, for example, change in pressure is considerable. These results suggest that such abiotic change may drive variation between fixed and unfixed samples.

In summary, it is likely that: (i) fixation vs RNA loss dynamics and (ii) community change during sampling are playing a role in driving the observed community changes and indeed these phenomena are not mutually exclusive. Depending on the aim of a given study, in situ preservation could be interpreted as a weakness as it limits the diversity profile recovered. Yet, in situ preserved samples are likely to recover a more realistic RNA community structure as a product of blocking RNA degradation or community change during sampling. As such, experimental design, with-or-without in situ RNA preservation, should be tailored to the specific aim of a study. We also note that overall the *LG*-*in situ* and *LG*-*ship* samples showed considerable community similarity, demonstrating that on-ship RNA fixation is still a valid approach for understanding eukaryotic microbial community.

### The community structure identified in the dark sediments

Our analysis identified a large relative abundance of rRNA sequences classified as diatoms. This pattern was consistent over multiple cores/sites. The extent of this rRNA signature was unexpected given that diatoms predominantly function as phototrophic organisms. Export of organic carbon to the deep sea is known to occur episodically and rapidly, in the order of days, in eastern North Pacific waters South of our study region [60], and sinking of diatoms has largely been attributed to mass aggregation during bloom senescence [61]. Further, a study from the western South Atlantic posited that phytoplankton export mechanisms included both transport as aggregates (or in faecal pellets) and sinking of individual living diatom cells [45]. Physical processes such as eddy-related filament development have also been shown to bring particulate organic carbon, including cells, to depth rapidly and in a living state (without mass aggregation) [62]. Diatoms are also known to form a resting state and may survive for decades in the dark until they are remixed to the surface [63] and prior molecular diversity studies have reported rRNA gene sequences from eukaryotic phytoplankton in marine sediments [9]. The detection of ribosomally active diatoms in the marine sediments indicates that these cells are not dead and therefore potentially constituent a ‘seedbank’. Future phytoplankton export models must therefore account for a process where viable diatom cells can potentially re-enter sunlit upper-water column habitats [45, 64].

### Osmotrophic–lysotrophic–saprotrophic eukaryotic communities in marine sediments

Fungi are primarily osmotrophic and often encode potent lysotrophic functions [11], making ‘a living’ by secreting enzymes that breakdown complex and recalcitrant macromolecules, and taking up the resulting liberated metabolites. This process is important for detrital/saprotrophic function and the recycling of biological material in terrestrial environments. However, in contrast to some studies, we did not recover a large diversity or relative abundance of Dikarya fungi [6, 9, 14, 16, 65]. Our opisthokont phylogenetic analysis indicates that there are surprisingly few fungi present. This is contradictory to terrestrial saprotrophic/detrital environments where fungi dominate [66, 67]. This result could be a product of our cell lysis methodology, which may have failed to effectively disrupt fungal cell wall structures. However, our extraction protocol included a physical cell disruption step and a freeze-thaw cycle. In contrast to the absence of Fungi, we did find a very large diversity of the *Fonticula-*like protist lineages, suggesting this group, which branches with organisms that form slime-mould cellular structures, constitutes an important component of marine sedimentary communities [39].

The analysis of MAST groups demonstrated that a number of these protist phylogroups, initially identified in the marine water column [44], are also ribosomally active in marine sediments. These data (combined with recent results [37, 51]) suggest that several MAST groups, specifically MAST-6, play a role in marine sediments—possibly as colonisers of other species which have fallen out of the water column, OLS agents or predators. Interestingly, it has been shown that some MAST groups live on diatoms [68]. It is therefore possible that a subset of the MAST groups could represent parasites.

Collectively, the pattern of OTU diversity identified demonstrates a number of candidate phylogenetic groups, specifically: pseudofungi, labyrinthulomycetes and fonticulids, which are ribosomally active, diverse and in some cases show high SSU rRNA relative abundances in these environments. It is not possible to confidently predict the function of these eukaryotic microbes based on SSU rRNA phylogenetic associations, yet the diversity pattern reported provides a number of candidate groups that potentially encode OLS function associated with detrital processing in marine sediments (Fig. 3c). However, it is important to note that many of these groups can have dual functions (osmotrophy and phagotrophy) e.g. the labyrinthulomycetes [69–71]. Indeed, multiple trophic functions are likely to be displayed by slime moulds (e.g. the *Fonticula-*like groups identified). The patterns identified here suggest these groups may be important organic matter degraders. If this is the case, it is likely that these unicellular eukaryotes are therefore adding to key biogeochemical functions.

## Supplementary information

Supplementary Materials Figure Legends

Supplementary Film S1

Supplementary Figure S1

Supplementary Figure S2

Supplementary Figure S3

Supplementary Figure S4

Supplementary Table S1

Supplementary Table S2

## References

[1] Ciais P, Sabine C, Bala G, Bopp L, Brovkin V, Canadell J, et al. Carbon and other biogeochemical cycles. In: Stocker TF, Qin D, Plattner G-K, Tignor M, Allen SK, Boschung J, et al., editors. Climate change 2013: the physical science basis. Contribution of Working Group I to the Fifth Assessment Report of the Intergovernmental Panel on Climate Change. Cambridge University Press, Cambridge, UK and New York, NY, USA; 2013.

[2] Hedges JI, Keil RG (1995). Sedimentary organic matter preservation: an assessment and speculative synthesis. Mar Chem.

[3] Jørgensen BB, Boetius A (2007). Feast and famine—microbial life in the deep-sea bed. Nat Rev Microbiol.

[4] Orcutt BN, Sylvan JB, Knab NJ, Edwards KJ (2011). Microbial ecology of the dark ocean above, at, and below the seafloor. Microbiol Mol Biol Rev.

[5] Lloyd KG, Schreiber L, Petersen DG, Kjeldsen KU, Lever MA, Steen AD (2013). Predominant archaea in marine sediments degrade detrital proteins. Nature.

[6] Edgcomb VP, Kysela DT, Teske A, de Vera Gomez A, Sogin ML (2002). Benthic eukaryotic diversity in the Guaymas Basin hydrothermal vent environment. Proc Natl Acad Sci.

[7] Lopez-Garcia P, Philippe H, Gail F, Moreira D (2003). Autochthonous eukaryotic diversity in hydrothermal sediment and experimental microcolonizers at the Mid-Atlantic Ridge. Proc Natl Acad Sci.

[8] Takishita K, Yubuki N, Kakizoe N, Inagaki Y, Maruyama T (2007). Diversity of microbial eukaryotes in sediment at a deep-sea methane cold seep: surveys of ribosomal DNA libraries from raw sediment samples and two enrichment cultures. Extremophiles.

[9] Orsi W, Biddle JF, Edgcomb V (2013). Deep sequencing of subseafloor eukaryotic rRNA reveals active fungi across marine subsurface provinces. PLoS One.

[10] Bik HM, Sung W, De Ley P, Baldwin JG, Sharma J, Rocha-Olivares A (2012). Metagenetic community analysis of microbial eukaryotes illuminates biogeographic patterns in deep-sea and shallow water sediments. Mol Ecol.

[11] Richards TA, Talbot NJ (2018). Osmotrophy. Curr Biol.

[12] Worden AZ, Follows MJ, Giovannoni SJ, Wilken S, Zimmerman aE, Keeling PJ (2015). Rethinking the marine carbon cycle: factoring in the multifarious lifestyles of microbes. Science.

[13] Orsi WD, Richards TA, Francis WR (2018). Predicted microbial secretomes and their target substrates in marine sediment. Nat Microbiol.

[14] Edgcomb VP, Beaudoin D, Gast R, Biddle JF, Teske A (2011). Marine subsurface eukaryotes: the fungal majority. Environ Microbiol.

[15] Richards TA, Leonard G, Mahé F, del Campo J, Romac S, Jones MDM (2015). Molecular diversity and distribution of marine fungi across 130 European environmental samples. Proc R Soc B Biol Sci.

[16] Burgaud G, Woehlke S, Rédou V, Orsi W, Beaudoin D, Barbier G (2013). Deciphering the presence and activity of fungal communities in marine sediments using a model estuarine system. Aquat Micro Ecol.

[17] Pawlowski J, Christen R, Lecroq B, Bachar D, Shahbazkia HR, Amaral-Zettler L (2011). Eukaryotic richness in the abyss: insights from pyrotag sequencing. PLoS ONE.

[18] Corinaldesi C, Barucca M, Luna GM, Dell’anno A (2011). Preservation, origin and genetic imprint of extracellular DNA in permanently anoxic deep-sea sediments. Mol Ecol.

[19] Rissanen AJ, Kurhela E, Aho T, Oittinen T, Tiirola M (2010). Storage of environmental samples for guaranteeing nucleic acid yields for molecular microbiological studies. Appl Microbiol Biotechnol.

[20] Mahé F, Mayor J, Bunge J, Chi J, Siemensmeyer T, Stoeck T (2015). Comparing high-throughput platforms for sequencing the V4 region of SSU-rDNA in environmental microbial eukaryotic diversity surveys. J Eukaryot Microbiol.

[21] Spain AM, Elshahed MS, Najar FZ, Krumholz LR (2015). Metatranscriptomic analysis of a high-sulfide aquatic spring reveals insights into sulfur cycling and unexpected aerobic metabolism. PeerJ.

[22] Lay CY, Mykytczuk NCS, Yergeau É, Lamarche-Gagnon G, Greer CW, Whyte LG (2013). Defining the functional potential and active community members of a sediment microbial community in a high-arctic hypersaline subzero spring. Appl Environ Microbiol.

[23] Takishita K, Kakizoe N, Yoshida T, Maruyama T (2010). Molecular evidence that phylogenetically diverged ciliates are active in microbial mats of deep-sea cold-seep sediment. J Eukaryot Microbiol.

[24] Bernhard JM, Kormas K, Pachiadaki MG, Rocke E, Beaudoin DJ, Morrison C (2014). Benthic protists and fungi of Mediterranean deep hypsersaline anoxic basin redoxcline sediments. Front Microbiol.

[25] Stoeck T, Bass D, Nebel M, Christen R, Jones MDM, Breiner H-W (2010). Multiple marker parallel tag environmental DNA sequencing reveals a highly complex eukaryotic community in marine anoxic water. Mol Ecol.

[26] Zhang J, Kobert K, Flouri T, Stamatakis A (2014). PEAR: a fast and accurate Illumina Paired-End reAd mergeR. Bioinformatics.

[27] Martin M (2011). Cutadapt removes adapter sequences from high-throughput sequencing reads. EMBnet J.

[28] Rognes T, Flouri T, Nichols B, Quince C, Mahé F (2016). VSEARCH: a versatile open source tool for metagenomics. PeerJ.

[29] Mahé F, Rognes T, Quince C, de Vargas C, Dunthorn M (2015). Swarm v2: highly-scalable and high-resolution amplicon clustering. PeerJ.

[30] Guillou L, Bachar D, Audic S, Bass D, Berney C, Bittner L (2013). The Protist Ribosomal Reference database (PR2): a catalog of unicellular eukaryote Small Sub-Unit rRNA sequences with curated taxonomy. Nucleic Acids Res.

[31] Edgar RC, Haas BJ, Clemente JC, Quince C, Knight R (2011). UCHIME improves sensitivity and speed of chimera detection. Bioinformatics.

[32] R Core Team. R: a language and environment for statistical computing. Vienna, Austria: R Foundation for Statistical Computing; 2014. https://www.R-project.org/.

[33] Wickham H (2017). ggplot2—elegant graphics for data analysis (2nd Edition). J Stat Softw.

[34] Lozupone C, Lladser ME, Knights D, Stombaugh J, Knight R (2011). UniFrac: an effective distance metric for microbial community comparison. ISME J.

[35] Katoh K, Standley DM (2013). MAFFT multiple sequence alignment software version 7: improvements in performance and usability. Mol Biol Evol.

[36] Venables WN, Ripley BD. Modern applied statistics with S. 4th edition. New York: Springer; 2002. ISBN 0-387-95457-0.

[37] Massana R, del Campo J, Sieracki ME, Audic S, Logares R (2014). Exploring the uncultured microeukaryote majority in the oceans: reevaluation of ribogroups within stramenopiles. ISME J.

[38] Pan J, del Campo J, Keeling PJ (2017). Reference tree and environmental sequence diversity of labyrinthulomycetes. J Eukaryot Microbiol.

[39] del Campo J, Mallo D, Massana R, de Vargas C, Richards TA, Ruiz-Trillo I (2015). Diversity and distribution of unicellular opisthokonts along the European coast analysed using high-throughput sequencing. Environ Microbiol.

[40] Stamatakis A (2006). RAxML-VI-HPC: maximum likelihood-based phylogenetic analyses with thousands of taxa and mixed models. Bioinformatics.

[41] Miller MA, Pfeiffer W, Schwartz T. Creating the CIPRES Science Gateway for inference of large phylogenetic trees. 2010 Gateway Computing Environments Workshop (GCE). New Orleans, LA 2010. p. 1–8.

[42] Moon-van der Staay SY, De Wachter R, Vaulot D (2001). Oceanic 18S rDNA sequences from picoplankton reveal unsuspected eukaryotic diversity. Nature.

[43] López-Garcí aP, Rodríguez-Valera F, Pedrós-Alió C, Moreira D (2001). Unexpected diversity of small eukaryotes in deep-sea Antarctic plankton. Nature.

[44] Massana R, Castresana J, Balague V, Guillou L, Romari K, Valentin K (2004). Phylogenetic and ecological analysis of novel marine stramenopiles. Appl Environ Microbiol.

[45] Durkin CA, Van Mooy BAS, Dyhrman ST, Buesseler KO (2016). Sinking phytoplankton associated with carbon flux in the Atlantic Ocean. Limnol Oceanogr.

[46] Suzuki N, Not F. Biology and ecology of radiolaria. In: Ohtsuka S, Suzaki T, Horiguchi T, Suzuki N, Not F, editors. Marine protists. Springer, Tokyo, Japan; 2015. p. 179–222.

[47] Torruella G, De Mendoza A, Grau-Bové X, Antó M, Chaplin MA, Del Campo J (2015). Phylogenomics reveals convergent evolution of lifestyles in close relatives of animals and fungi. Curr Biol.

[48] Cavalier-Smith T. The origin of Fungi and pseudofungi. In: Rayner ADM, Brasier CM, Moore D, editors. Evol Biol Fungi. Cambridge University Press. 1987;12:339–53.

[49] Raghukumar S, Ramaiah N, Raghukumar C (2001). Dynamics of thraustochytrid protists in the water column of the Arabian sea. Aquat Micro Ecol.

[50] Raghukumar S, Sharma S, Raghukumar C, Sathe-Pathak V, Chandramohan D (1994). Thraustochytrid and fungal component of marine detritus. IV. Laboratory studies on decomposition of leaves of the mangrove *Rhizophora apiculata* Blume. J Exp Mar Bio Ecol.

[51] Logares R, Audic S, Santini S, Pernice MC, de Vargas C, Massana R (2012). Diversity patterns and activity of uncultured marine heterotrophic flagellates unveiled with pyrosequencing. ISME J.

[52] Leonard G, Labarre A, Milner DS, Monier A, Soanes D, Wideman JG (2018). Comparative genomic analysis of the ‘pseudofungus’ *Hyphochytrium catenoides*. Open Biol.

[53] Collado-Mercado E, Radway JC, Collier JL (2010). Novel uncultivated labyrinthulomycetes revealed by 18S rDNA sequences from seawater and sediment samples. Aquat Micro Ecol.

[54] Worley AC, Raper KB, Hohl M (1979). *Fonticula alba*: a new cellular slime mold (Acrasiomycetes). Mycologia.

[55] Mendoza L, Taylor JW, Ajello L (2002). The class mesomycetozoea: a heterogeneous group of microorganisms at the animal-fungal boundary. Annu Rev Microbiol.

[56] Le Calvez T, Burgaud G, Mahé S, Barbier G, Vandenkoornhuyse P (2009). Fungal diversity in deep-sea hydrothermal ecosystems. Appl Environ Microbiol.

[57] Stewart FJ, Dalsgaard T, Young CR, Thamdrup B, Revsbech NP, Ulloa O (2012). Experimental incubations elicit profound changes in community transcription in OMZ bacterioplankton. PLoS ONE.

[58] Massana R, Pedrόs-Aliό C, Casamayor EO, Gasol JM (2001). Changes in marine bacterioplankton phylogenetic composition during incubations designed to measure biogeochemically significant parameters. Limnol Oceanogr.

[59] Edgcomb VP, Taylor C, Pachiadaki MG, Honjo S, Engstrom I, Yakimov M (2016). Comparison of Niskin vs. in situ approaches for analysis of gene expression in deep Mediterranean Sea water samples. Deep Sea Res Part II Top Stud Oceanogr.

[60] Smith KL, Huffard CL, Kahru M, Messié M, Ruhl HA (2018). Episodic organic carbon fluxes from surface ocean to abyssal depths during long-term monitoring in NE Pacific. Proc Natl Acad Sci.

[61] Alldredge AL, Gotschalk C, Passow U, Riebesell U (1995). Mass aggregation of diatom blooms: insights from a mesocosm study. Deep Sea Res Part II Top Stud Oceanogr.

[62] Omand MM, D’Asaro EA, Lee CM, Perry MJ, Briggs N, Cetini I (2015). Eddy-driven subduction exports particulate organic carbon from the spring bloom. Science.

[63] Kamp A, de Beer D, Nitsch JL, Lavik G, Stief P (2011). Diatoms respire nitrate to survive dark and anoxic conditions. Proc Natl Acad Sci.

[64] Simmons MP, Bachy C, Sudek S, van Baren MJ, Sudek L, Ares M (2015). Intron invasions trace algal speciation and reveal nearly identical Arctic and Antarctic *Micromonas* populations. Mol Biol Evol.

[65] Takishita K, Tsuchiya M, Reimer JD, Maruyama T (2006). Molecular evidence demonstrating the basidiomycetous fungus *Cryptococcus curvatus* is the dominant microbial eukaryote in sediment at the Kuroshima Knoll methane seep. Extremophiles.

[66] Tedersoo L, Bahram M, Polme S, Koljalg U, Yorou NS, Wijesundera R (2014). Global diversity and geography of soil fungi. Science.

[67] O’Brien HE, Parrent JL, Jackson JA, Moncalvo J-M, Vilgalys R (2005). Fungal community analysis by large-scale sequencing of environmental samples. Appl Environ Microbiol.

[68] Gómez F, Moreira D, Benzerara K, López-Garcí aP (2011). Solenicola setigera is the first characterized member of the abundant and cosmopolitan uncultured marine stramenopile group MAST-3. Environ Microbiol.

[69] Raghukumar S (1992). Bacterivory: a novel dual role for thraustochytrids in the sea. Mar Biol.

[70] Raghukumar S (2002). Ecology of the marine protists, the labyrinthulomycetes (thraustochytrids and labyrinthulids). Eur J Protistol.

[71] Tsui CKM, Marshall W, Yokoyama R, Honda D, Lippmeier JC, Craven KD (2009). Labyrinthulomycetes phylogeny and its implications for the evolutionary loss of chloroplasts and gain of ectoplasmic gliding. Mol Phylogenet Evol.

