# Supplementary material for: Controlled sampling of ribosomally active protistan diversity in sediment-surface layers identifies putative players in the marine carbon sink
Source: ISME J. 2020 Jan 9;14(4):984–98. doi: 10.1038/s41396-019-0581-y (PMC7082347; doi:10.1038/s41396-019-0581-y)
Supplement: Supplementary file 1 — Supplementary Materials Figure Legends [file 41396_2019_581_MOESM1_ESM.docx]

**Supplementary Materials Legends**

**Supplementary Figure S1. UPGMA clustering comparing push cores/sampling methods and sample replicates**. From Bray Curtis distance analysis; A) Comparison of OTU presence-absence data, and B) Comparison of OTU Relative abundance data. From Unifrac distance analysis; C) Unweighted (Qualitative), and D) Weighted (Quantitative) comparisons.

**Supplementary Figure S2. Phylogenetic tree of the stramenopiles**. Detailed phylogram of tree shown in figure 4a. OTUs from this study appear in red. Previously released database sequences are shown in black. ML bootstrap values higher than 50% are shown. Heatmaps aligned with the taxon labels on the tree illustrate the number or reads per core.

**Supplementary Figure S3. Phylogeny of Labyrinthulomycetes (Labyrinthulea).** Detailed phylogram of tree shown in figure 4b. OTUs from this study appear in red. Previously released database sequences are shown in black. ML bootstrap values higher than 50% are shown. Heatmaps aligned with the taxon labels illustrate the number or reads per sampling core.

**Supplementary Figure S4. Phylogeny of the opisthokonts (excluding metazoan).** Detailed phylogram of tree shown in figure 4c. OTUs from this study appear in red. Previously released database sequences are shown in black. ML bootstrap values higher than 50% are shown. Heatmaps aligned with the taxon labels illustrate the number or reads per core.

**Supplementary Table S1.** Number of reads per replicate after bioinformatic processing and before rarefaction analysis. Aa-Cg correspond to replicate codes given in figure 1C.

**Supplementary Table S2.** % relative abundance of OTUs of putative eukaryotic phytoplankton affiliation.

**Supplementary Film S1.** Demonstration of deployment of deep-sea RNA buffer injector - core system.
