# Supplementary material for: Controlled sampling of ribosomally active protistan diversity in sediment-surface layers identifies putative players in the marine carbon sink
Source: ISME J. 2020 Jan 9;14(4):984–98. doi: 10.1038/s41396-019-0581-y (PMC7082347; doi:10.1038/s41396-019-0581-y)
Supplement: Supplementary file 3 — Supplementary Figure S1 [file 41396_2019_581_MOESM3_ESM.pdf]

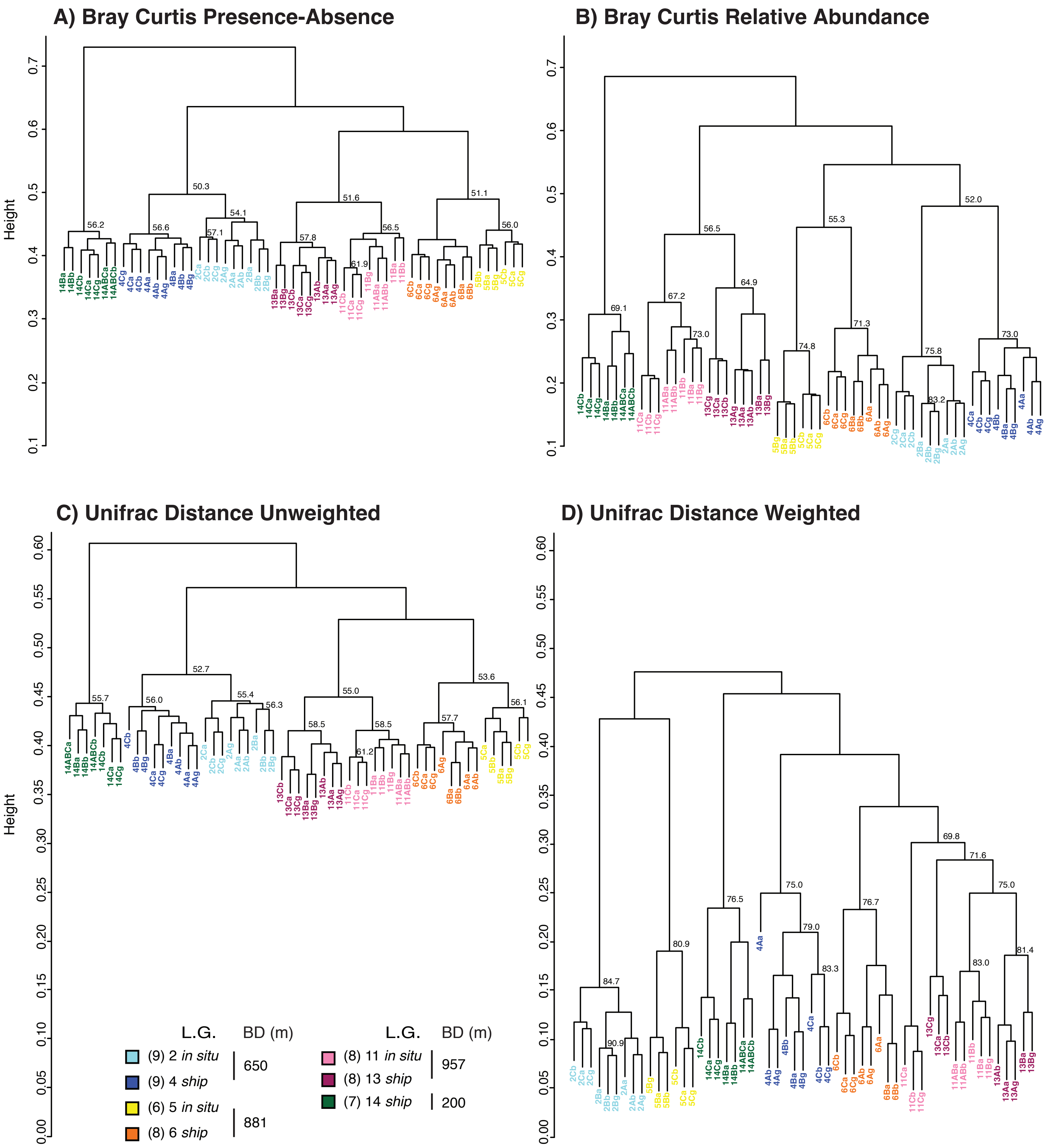
A) Bray Curtis Presence-Absence
B) Bray Curtis Relative Abundance
C) Unifrac Distance Unweighted
D) Unifrac Distance Weighted
Height
L.G.
BD (m)
(9) 2 in situ
(9) 4 ship
650
(6) 5 in situ
(8) 6 ship
881
(8) 11 in situ
(8) 13 ship
957
(7) 14 ship
200
