# Supplementary figures and images for: Controlled sampling of ribosomally active protistan diversity in sediment-surface layers identifies putative players in the marine carbon sink
Source: ISME J. 2020 Jan 9;14(4):984–98. doi: 10.1038/s41396-019-0581-y (PMC7082347; doi:10.1038/s41396-019-0581-y)

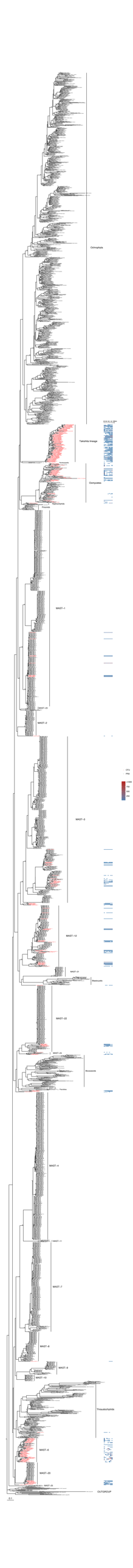

Supplement: Supplementary file 4 — Supplementary Figure S2 [file 41396_2019_581_MOESM4_ESM.pdf]

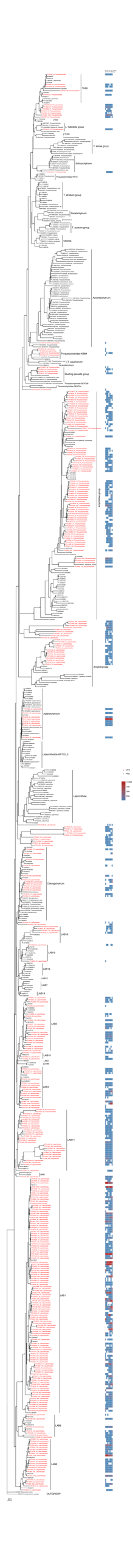

Supplement: Supplementary file 5 — Supplementary Figure S3 [file 41396_2019_581_MOESM5_ESM.pdf]
