# Supplementary material for: Controlled sampling of ribosomally active protistan diversity in sediment-surface layers identifies putative players in the marine carbon sink
Source: ISME J. 2020 Jan 9;14(4):984–98. doi: 10.1038/s41396-019-0581-y (PMC7082347; doi:10.1038/s41396-019-0581-y)
Supplement: Supplementary file 7 — Supplementary Table S1 [file 41396_2019_581_MOESM7_ESM.pdf]

RUN 1

	core 2	core 4	core 5	core 6	core 11	core 13	core 14
Aa	348225	92780	193389	111372	29928	101639	72428
Aa extra							
Ab	125981	98737	131787	114048	41340	85603	66854
Ab extra							
Ag	116094	86695	147119	85111		120659	
Ag extra							
Total A	**590300**	**278212**	**472295**	**310531**	**71268**	**307901**	**139282**
Ba	94350	92200		130838	19454	62158	67053
Ba extra							
Bb	169999	94748		97394	20980		71319
Bb extra							
Bg	97040	61214			31507	64864	110294
Bg extra							
Total B	**361389**	**248162**		**228232**	**71941**	**127022**	**248666**
Ca	60586	70753	47534	66755	101562	118697	51173
Ca extra							
Cb	104330	114886	117038	84208	106651	74622	
Cb extra							
Cg	44169	73589	128486	84282	121849	77568	116024
Cg extra							
Total C	**209085**	**259228**	**293058**	**235245**	**330062**	**270887**	**167197**
Total cores	**1160774**	**785602**	**765353**	**774008**	**473271**	**705810**	**555145**
						Total reads	**5219963**

RUN1 + RUN2

	core 2	core 4	core 5	core 6	core 11	core 13	core 14
Aa	348339	92857	193452	111440	29964	101750	72576
Aa extra		224968		261555		245438	184892
Ab	126035	98828	131834	114112	41385	85689	66994
Ab extra		232031		259342		204807	162356
Ag	116134	86767	147173	85160		120749	
Ag extra		209331		198226		292018	232713
Total A	**590508**	**944782**	**472459**	**1029835**	**71349**	**1050451**	**719531**
Ba	94386	92282		130943	19473	62223	67208
Ba extra		221744		299580		151845	165388
Bb	170066	94833		97481	20998		71471
Bb extra		222329		231763		190209	172854
Bg	97070	61276			31535	64929	
Bg extra		147098		158193		152535	
Total B	**361522**	**839562**		**917960**	**72006**	**621741**	**476921**
Ca	60618	70859	47547	66812	101610	118797	110520
Ca extra		171071		162422		286205	271896
Cb	104372	115004	117098	84269	106708	74679	51272
Cb extra		278884		195504		177696	123666
Cg	44197	73653	128536	84331	121916	77638	116268
Cg extra		179090		198552		186792	277942
Total C	**209187**	**888561**	**293181**	**791890**	**330234**	**921807**	**951564**
Total cores	**1161217**	**2672905**	**765640**	**2739685**	**473589**	**2593999**	**2148016**
						Total reads	**12555051**
