# Supplementary material for: Controlled sampling of ribosomally active protistan diversity in sediment-surface layers identifies putative players in the marine carbon sink
Source: ISME J. 2020 Jan 9;14(4):984–98. doi: 10.1038/s41396-019-0581-y (PMC7082347; doi:10.1038/s41396-019-0581-y)
Supplement: Supplementary file 8 — Supplementary Table S2 [file 41396_2019_581_MOESM8_ESM.pdf]

**Phytoplankton**	**core14**	**core2**	**core4**	**core5**	**core6**	**core11**	**core13**
Bacillariophyta	14.4365684	28.4219017	24.2195237	37.0623051	17.0105302	17.5232111	11.9840199
Bolidophyceae-and-relatives	0.13450481	0.11043331	0.15963209	0.14886326	0.19616165	0.16469977	0.17187899
Chrysomerophyceae	0	0	0	0	0.00021115	0	0
Chrysophyceae-Synurophyceae	0.47720703	0.40604828	0.30490573	0.52999542	0.3595945	0.64739679	0.58848495
Dictyochophyceae	0.02512727	0.0118246	0.0268165	0.19890665	0.21981085	0.0295615	0.03209534
Eustigmatophyceae	0.00253384	0.00211154	0.00232269	0.00105577	0.00802384	0.01541421	0.01245806
MOCH	0.05701146	0.04201956	0.08192758	0.08150527	0.08066066	0.08087181	0.09649717
Ochrophyta_X	0.00253384	0	0.00021115	0	0	0	0
Pelagophyceae	0.01520306	0.10325409	0.08868449	0.10536562	0.10874408	0.0804495	0.10642139
Phaeophyceae	0.00443423	0.00105577	0.00126692	0.00168923	0.00548999	0.00168923	0.00211154
Phaeothamniophyceae	0.00506769	0.00021115	0.00021115	0.00042231	0.00063346	0	0.00084461
Raphidophyceae	0.00485653	0	0.00084461	0.00168923	0.00253384	0.00063346	0.00042231
Xanthophyceae	0	0	0	0	0	0.00021115	0.00042231
Dinophyceae	3.28660505	1.60033278	2.02137296	2.05431292	2.13919665	3.05349153	2.85162873
Dinophyta_X	0.01076883	0.02111536	0.00886845	0.00401192	0.0086573	0.0086573	0.0295615
