# Supplementary material for: Controlled sampling of ribosomally active protistan diversity in sediment-surface layers identifies putative players in the marine carbon sink
Source: ISME J. 2020 Jan 9;14(4):984–98. doi: 10.1038/s41396-019-0581-y (PMC7082347; doi:10.1038/s41396-019-0581-y)
Supplement: Supplementary file 6 — Supplementary Figure S4 [file 41396_2019_581_MOESM6_ESM.pdf]

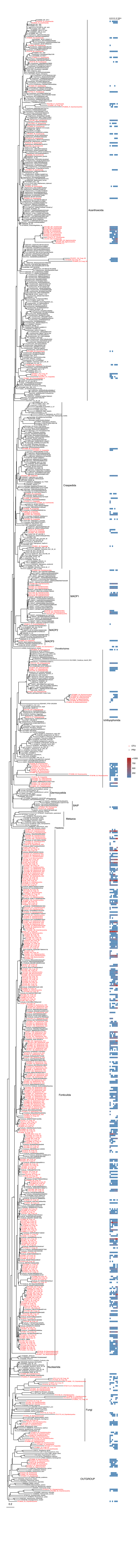

Acanthoecida
Craspedida
MAOP1
MAOP2
MAOP3
Ichthyophonida
Metazoa
Fonticulida
Nucleariida
Fungi
OUTGROUP
0.2
